# Emulation with Organic Memristive Devices of Impairment of LTP Mechanism in Neurodegenerative Disease Pathology

**DOI:** 10.1155/2017/6090312

**Published:** 2017-06-19

**Authors:** Silvia Battistoni, Victor Erokhin, Salvatore Iannotta

**Affiliations:** ^1^IMEM-CNR, 43124 Parma, Italy; ^2^Institute of Fundamental Medicine and Biology, Kazan Federal University, Kazan, Russia

## Abstract

We explore and demonstrate the extension of the synapse-mimicking properties of memristive devices to a dysfunctional synapse as it occurs in the Alzheimer's disease (AD) pathology. The ability of memristive devices to reproduce synapse properties such as LTP, LTD, and STDP has been already widely demonstrated, and moreover, they were used for developing artificial neuron networks (perceptrons) able to simulate the information transmission in a cell network. However, a major progress would be to extend the common sense of neuromorphic device even to the case of dysfunction of natural synapses. Can memristors efficiently simulate them? We provide here evidences of the ability of emulating the dysfunctional synaptic behavior typical of the AD pathology with organic memristive devices considering the effect of the disease not only on a single synapse but also in the case of a neural network, composed by numerous synapses.

## 1. Introduction

The increase in life expectancy during the 20th century has resulted in a rising number of reported cases of individuals achieving one of the age-related neurodegenerative disorders among which one of the most studied and invalidating disorder is the Alzheimer's disease [1].

Macroscopic and behavioral alterations include progressive memory impairment, paranoia, delusions, and loss of social appropriateness and a progressive decline in language and cognitive function [1]. From the microscopic point of view, this disease induces the formation of 2 classes of lesions in neurons: senile neurotic plaques that contain extracellular deposits of amyloid *β*-protein (A*β*) and neurofibrillary tangles composed of the microtubule-associated protein tau. These two lesions are coupled with the decrease in the amounts of acetylcholine and activities of the synthetic and degradative enzymes, choline acetyltransferase, and acetylcholinesterase [1, 2].

Further studies reported that the Alzheimer's disease is always accompanied, from a cellular point of view, with deficits in several neurotransmitters (corticotropin-releasing factor, somatostatin, GABA, and serotonin), and the early symptoms seem to be correlated with dysfunction of cholinergic and glutamatergic synapses and of the enzymes that generate and metabolize acetylcholine [1, 3]. What appears clear in this scenario is the intimate connection between the initial phase of the disease and the synapses involved that, during the progress of the syndrome, decrease in numerical density and undergo changes in the presynaptic vesicle protein synaptophysin in the hippocampus and association cortices [1, 3]. Moreover, significant deficits in basal synaptic transmission and/or long-term potentiation were reported even well before the appearance of the early symptoms and plaques formation [1, 3, 4]. Different groups reported the effect of different mutations of the amyloid *β* protein precursor (APP) on the synaptic plasticity suggesting that the changes that the synapses undergo during the disease's progress are functional (due to the progressive impairment of the performance in a spatial working memory task and smaller excitatory postsynaptic potentials) or structural (due to a significant reduction in synaptic number and density) [1, 3, 5]. In these cases, the ability of the synapses to perform the LTP mechanism and a normal basal transmission is compromised.

Oddo et al. [6] demonstrated that mice with triple-transgenic model (3xTg-AD) develop plaques and tangles typical of the Alzheimer's disease together with deficits in synaptic plasticity (LTP).

In the emerging field of the biomimicking electronics, memristors play a central role due to their ability of modulating the internal resistance as a function of the charge that passed through them [7]. This is why they represent good candidates for emulating different typical synapse mechanisms such as potentiation or depression of synaptic strength (LTP, LTD, and STDP) [8, 9].

Organic memristive devices were proposed in 2005 as hybrid electronic elements based on a polyaniline- (PANI-) polyethylene oxide (PEO) junction; the current-voltage characteristics of which were attributed to the electrochemical reactions of the PANI under the solid electrolyte [10]. This working principle was demonstrated through UV-vis and FTIR Raman spectroscopies and time-resolved X-ray fluorescence analysis [11, 12]. The role of the polyelectrolyte, composed by a polyethylene oxide matrix with lithium salt, was also investigated, concluding that the best performances of the device are obtained using a high-average molecular weight of the PEO and controlling the cation's nature and by the solution's pH [13].

These devices were used for developing adaptive networks in which the synaptic role is played by organic memristive devices since the modulation in the conduction properties emulates very well the synaptic behavior of induced changes in the signal propagation path in a real neuronal network [14–17].

Since then, such kinds of electrochemical elements are considered as polymeric analogues of the synaptic components of the nervous system, and this analogy was further demonstrated by the fabrication of an adaptive network realized by means of 8 devices all connected that presented an initial preferential conductive path, that was “rewritable” using an ad hoc training procedure [14, 15].

Recently, organic memristive devices have been used for the realization of elementary and bilayer perceptrons that are neural networks able to implement basic brain-inspired learning functionalities and parallel processing; moreover, they are able to solve classification tasks, for example, classification of linearly separable and nonseparable groups of objects [18, 19].

## 2. Materials and Methods

### 2.1. Device Preparation

For the device preparation, we used the same method reported in several papers [20–22]; briefly, a solution of PANI (Sigma-Aldrich Mw ≈ 10,000) was prepared in 1-methyl-2-pyrrolidinone (Sigma-Aldrich ACS reagent ≥99.0%) with a concentration of 0.1 mg mL^−1^ with the addition of 10% of Toluene (AnalaR NORMAPUR® ACS). The latter was spread on a water subphase and deposited on a glass substrate with two separated chrome electrodes using the Langmuir-Schaefer technique.

60 layers of this conductive polymer (PANI) form the active layer of the device in order to provide both a good conductivity and a fast diffusion process of the electrolyte ions into the PANI film. Since the deposition phase is performed using the emeraldine base form of the polymer, 2 doping processes are necessary to complete the protonation of the PANI into its conductive form (emeraldine salt). The doping processes were done by dipping the sample into HCl 1 M for 30 s and, after a resting time of 30–40 minutes, for 15 s.

We prepared a water solutions of polyethylene oxide with a molecular weight of 8 × 10^6^ Da (PEO) with a concentration of 20 mg ml^−1^, doped with 0.1 M LiClO_4_ (Sigma). This one was diluted in water with a ratio of 1 : 10 and divided in several preparations that differ one from the other by the addition of HCl 1 M inside (9, 4.5, 1.2, and 0%*v*/*v*). Since the electrolyte has been diluted, it was necessary to apply to the PANI-active layer a poly(ethylene-vinyl acetate) well containing the liquid and to fix the potential of the reference electrode (silver wire of 0.05 mm). The area of contact between the PANI layer and the electrolyte defined by the application of the well is considered as the ‘active zone', since all redox reactions and the conductivity variations occur there.

### 2.2. Device Characterization

For qualifying our devices, we applied voltage values between the Cr electrodes (Source-S- and Drain-D-) and we acquired two currents: the ionic current flowing through the reference electrode (gate current: IG) and the total current passing in the PANI channel between the S and D electrodes (drain current: ID). The application of the voltage and the measurements of the total current were performed with two source measure units (NI PXle 4138/9) and drove by an ad hoc LabVIEW code. For our experiment, we decided to use a pulse of 600 s during which we applied −0.2 V for 300 s and 0.8 V for the remaining 300 s, even if in our analysis, we consider just the second 300 s intervals; the initial application of a negative voltage is necessary for resetting of the system before a new cycle of measurement.

In order to avoid possible artifacts due to the eventual evaporation of the electrolyte during the measurements, we decided to change the liquid in the containing well regularly (every 1 or 2 measurements when the sequence of acquisitions is with the same electrolyte and every time in the case of different electrolytes).

The measurement routine consists of the following steps: we filled the well with the chosen electrolyte ensuring that it would fully cover the gate electrode and then we launched the scan. We repeated this routine with all the prepared electrolytes from the higher content of HCl to the 0%*v*/*v* one, carefully removing the already used electrolyte between one scan and the next one using a pipette. After the first acquisition with PEO solution without HCl, we decided to repeat it 3 times in order to “wash” the active zone of the device for removing all the residual Cl^−^ ions in the PANI film.

## 3. Results

For demonstrating the analogy to the synaptic failure typical of the Alzheimer's disease, we realized a memristive device using the same method reported in [21] and several polyelectrolyte solutions with different contents of HCl 1 M (from 9%*v*/*v* to 0%*v*/*v*). Reducing the amount of chloride ions inside the electrolyte leads to the increase of the total pH that varies from a strong acid to a neutral one. The polyaniline used for the fabrication of the device active channel is pH sensitive since [23] its conductive properties increase with the decreasing of this parameter [24].

As already mentioned, organic memristive devices show synaptic-mimicking properties being able to vary their internal resistance as a function of the previous activity of the device. In fact, using the polyelectrolyte with the higher content of HCl, it is possible to switch from high to low resistivity of the polymer in response of a proper mix of the voltage value and the application time.

**Figure 1:**
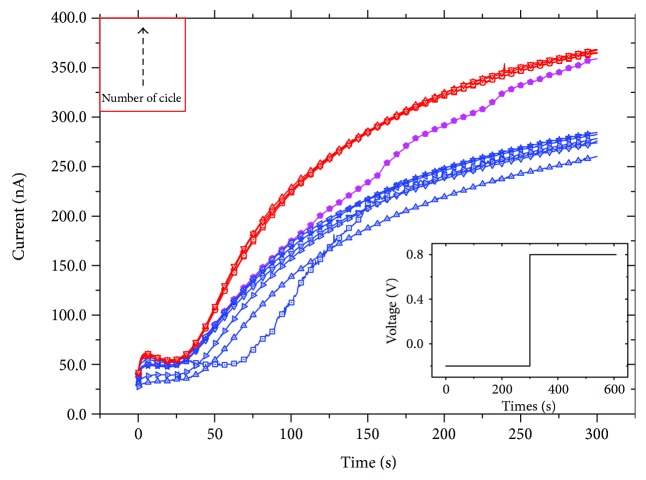
Demonstration of the LTP mechanism in the organic memristive devices: the blue curves are the initial responses, red ones are the final, and magenta is the transition curve. The profile of the given pulse is shown in the inset.

As reported in Figure 1, the application of 0.8 V (reported in inset) induces a gradual increase of the output current of the memristor that passes from 50 nA to 250 nA with a continuum of states. This initial consideration is already in good agreement with the reported synaptic LTP mechanism in which a proper set of stimuli produces a long-lasting increase of the synaptic strength [25]. Moreover, repeating this latter characterization, for a low number of measurements, all the current curves are mostly overlapped (blue curves in Figure 1), and they all reach a maximum value of more or less 250–260 nA.

**Figure 2:**
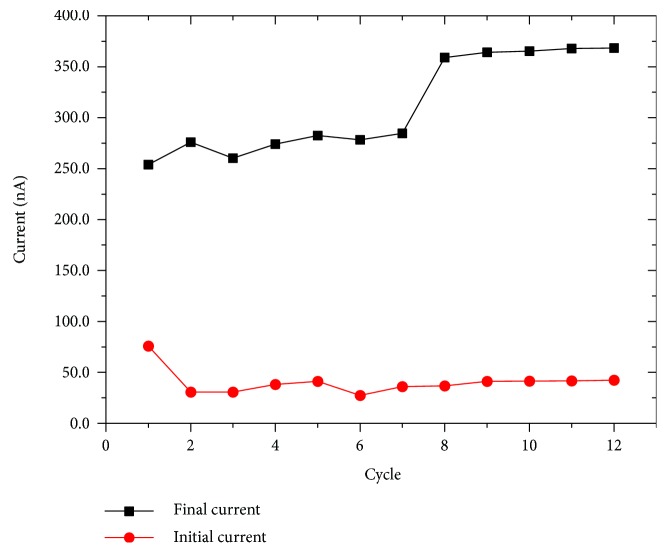
Demonstration of the LTP mechanism in the organic memristive devices: final and initial current value per number of the given pulse.

As the number of cycles increases, the enhancing trend of the curves remains the same but their maximum is shifted to higher values (350 nA) and, in general, the device shows a faster response to the voltage stimulus (red curves in Figure 1). In Figure 1, the transition curve (magenta) between the first and the second regime is also shown.  Figure 2 shows the maximum current (black curve) as a function of the number of cycles compared with the initial value (red curve). The initial values of the currents remain mostly constant for all the measurements while in the black curve, the two current regimes already described are evident.

The wide gap between the initial and the final current is typical for the organic memristor response and together with the capability of further increase, this gap is a function of the number (and the duration) of the stimuli. They constitute the strong parallelism between these device properties and the phenomenology of the typical LTP synaptic behavior.

As already mentioned, one of the early stages of the Alzheimer's disease is the impairment of the LTP transmission that results progressively less efficient and compromised.

In some cases, this effect is due to dysfunctional or structural reasons that undermine in one case the efficiency and in the other one the number and density of the involved synapses.

For this purpose, we realized a simple model for reproducing the same behavior also with memristive devices where the main role is played by the pH of the electrolyte solution: the impairment of the performances of the natural synapses will be represented here by a decreasing content of ions in the electrolytic preparation.

Thus, we tested our memristive device applying the same voltage pulse reported in Figure 1() but varying the electrolyte composition in order to simulate the progress of the disease.

**Figure 3:**
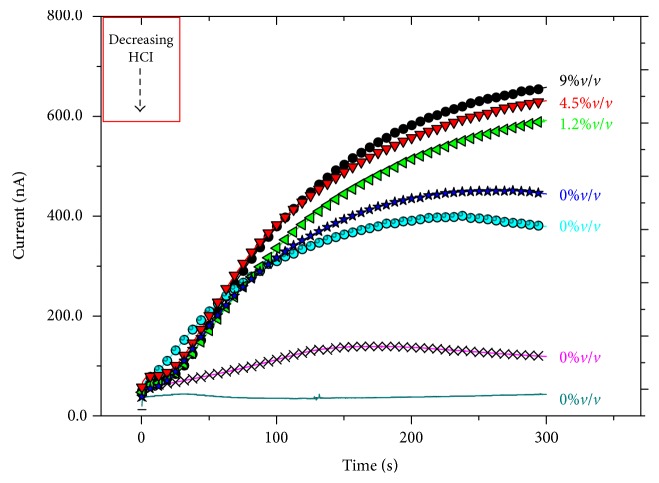
Memristor's responses to the voltage stimulus reported in the inset of Figure 1 for a progressive lowering of chloride ion content in the polyelectrolyte: black circles represent the curve obtained with the polyelectrolyte solution at 9%*v*/*v*; red triangles 4.5%*v*/*v*; green triangles 1.2%*v*/*v*; blue stars 0%*v*/*v*; light blues 0%*v*/*v* (1° wash); pink line 0%*v*/*v* (2° wash); and dark green 0%*v*/*v* (3° wash).

**Figure 4:**
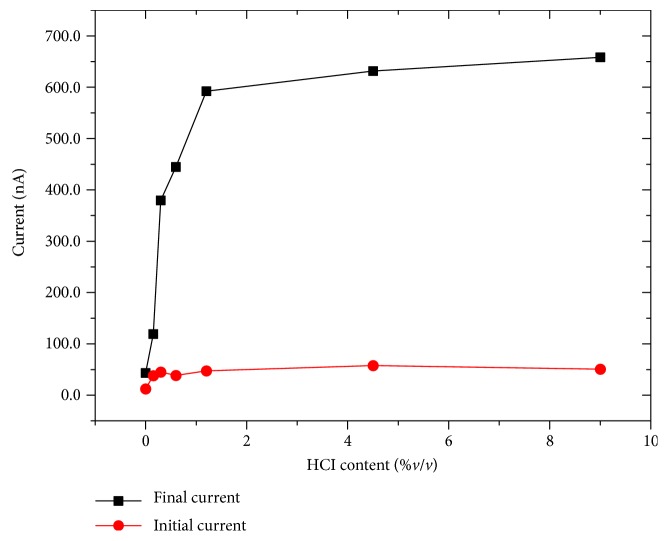
Final and initial current values of the Memristor's responses reported in Figure 3 for a progressive lowering of chloride ion content in the polyelectrolyte.

Results obtained (Figures 3 and 4) demonstrate that in our standard condition, (9%*v*/*v* di HCl) the device presents the widest difference between the initial and the final current values. This gap progressively decreases with decreasing acid content of the electrolyte even if the initial current is mostly reproducible for all the conditions. As expected, after the initial acquisition of the current output with a polyelectrolyte without HCl, it was necessary to perform some washing procedures to remove all the chloride ions from the PANI thin film. However, the potentiation effect of the voltage stimulus reduces progressively its efficiency as a function of the amount of Cl^−^ ions in the polyelectrolyte. This behavior is in good agreement with results obtained in the LTP tests reported by Oddo et al. [6].

However, it is to note that the progress of any kind of neurodegenerative disease is not characterized by an abrupt variation between the “working” and “not working” regimes, but it has a relatively slow progressive degeneration from the early stage to the final period of the disease.

This is partially due to the high plasticity and the adaptability of the neuronal network that is able to deal with a single synapse problem through compensation process [4]: this means that any dysfunctioning of a single synapse can be offset by the normal activity of another forthcoming cell.

**Figure 5:**
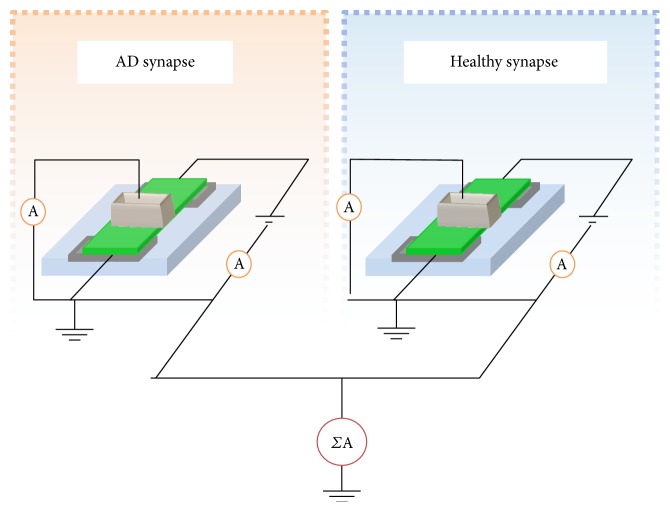
Scheme of the memristive system.

**Figure 6:**
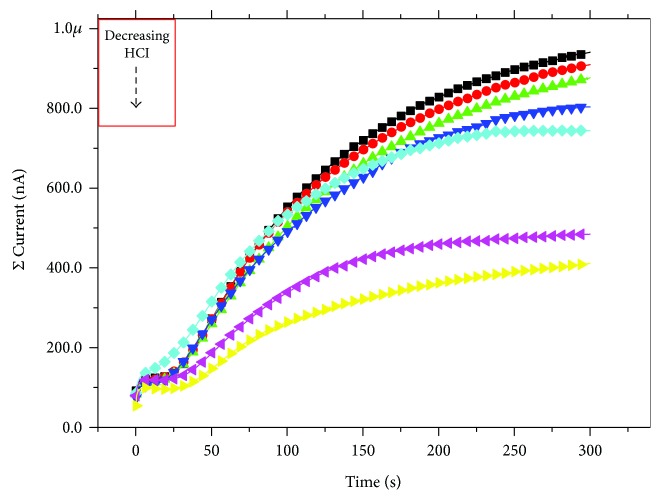
Memristor system's responses to the voltage stimulus reported in the inset of Figure 1 in which one device undergoes a performance degeneration inducted by a progressive lowering of chloride ion content in the polyelectrolyte: black squares represent the curve obtained in the 1° iteration; red circles 2° iteration; green triangles 3° iteration; blues triangles 4° iteration; light-blue squares 5° iteration; magenta triangles 6° iteration; and yellow triangles 7° iteration.

**Figure 7:**
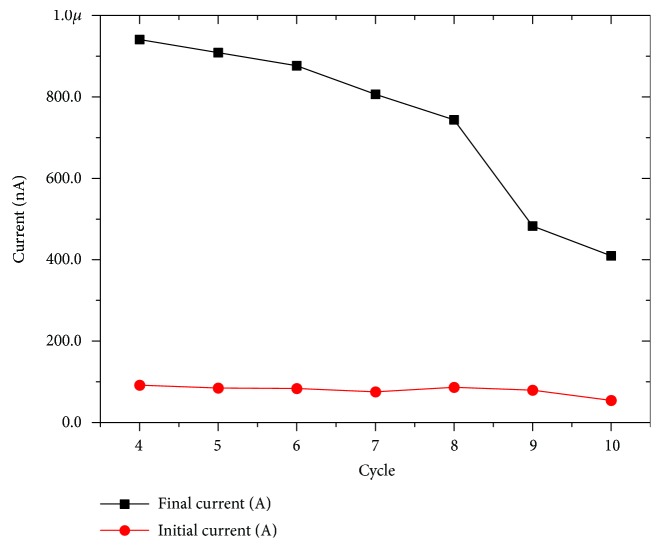
Final and initial current values of the Memristor system's responses reported in Figure 6 for a progressive lowering of chloride ion content in the polyelectrolyte.

In our simple model, we need to take this aspect into consideration and, thus, we decided to couple a performance of a healthy cell (for example the one reported in Figure 1) with the one in which AD disease occurs (Figure 3). A scheme of the system is reported in Figure 5, in which 2 memristive devices simulate an AD and a healthy synapse and the total system output results from the summation of the AD and not AD synapse current contributions. In this case, the application of 0.8 V as bias voltage for both the memristive devices gives the possibility of comparing and summing the two already obtained results (Figures 1 and 3) without the necessity of scaling factors. For this algebraic summation of the currents, we decided to use the entire set of curves of Figure 3 and seven curves of Figure 1 (from 4° to 10° iteration). Results, reported in Figures 6 and 7, demonstrate a less abrupt variation of the potentiation properties of the stimuli, as expected, and comparing the results of Figure 4 with the ones of Figure 7, the brusque variation of the final current curve of the first case is substituted with a more moderated variation due to the increasing contribution coming from the healthy synapse.

This softer and gradual decrease of the final current better emulates the realistic progress of the neurodegenerative disease in which the synaptic strength is progressively reduced from the early stage to the final period of the disease.

## 4. Conclusions

We reported for the first time on a neuromorphic system mimicking both the potentiation condition of a healthy synapse (LTP) and the progressive deficits of efficacy of a synapse affected by a neurodegenerative disease. The model we propose is rather simple, but nevertheless, it can take into account different effects typical for the AD disease such as the impairment of the efficiency of a synapse and the weighted reduction of the synaptic density. Moreover, increasing the number of devices involved and varying the number of synapses damaged by the disease in time can be extended. The suggested approach is ideally suitable for developing artificial systems over which experiment methods for pathology treatments. The next step will be to develop experiments where the device properties are recovered after aging by injection of lost ions, similarly to the approach used in Alzheimer's disease treatments.
